# A novel discovery model for revealing substitution relationships from international stock markets: With association rule analysis

**DOI:** 10.1016/j.heliyon.2024.e38774

**Published:** 2024-10-10

**Authors:** Luote Dai, Chengkui Huang, Chuyu Yu, Shengyu Gu

**Affiliations:** a School of Digital Economy & Trade, Wenzhou Polytechnic, Wenzhou, 325000, China; b Department of Business Administration, National Chung Cheng University, Chaiyi, Taiwan, 621301; c School of Geography and Tourism, Huizhou University, Huizhou, 516000, China

**Keywords:** International stock indices, Data mining, Association rule

## Abstract

At present, the trading volume of the stock market is huge, and the traditional method can not effectively find the relationship between the rise and fall of the stock market, but the machine learning method can find their interrelated data from a large number of data. This research aims to determine the effectiveness of association mining technology in analyzing the relationship between the ups and downs of stock markets in various countries, and it found the highest level of association between stock market items as investor references. The research data takes Taiwan's stock market as the target market and the international mainstream stock index as the related stock market. Through the analysis, it is found that association mining can accurately find the associated stock market according to the relevant parameters. The Taiwan stock market is more closely related to the top ten economies such as the Mainland, the United States, the United Kingdom and France, which shows that the rise of the international or mainland stock market will drive foreign capital to actively buy the Taiwan stock market, and vice versa. At last, the study sorted out three groups of stocks with the highest correlation degree according to the results of association mining, Namely Foxconn Stock (2354) and TSMC (2330), which are most closely related to the rise and fall of the international stock market. Therefore, the results of this study can also be used as a reference for investors to choose the stock price of Taiwan stock market.

## 1 Introduction

In the field of stock investment, predicting the future rise and fall of stocks has long challenged both practitioners and theorists [1]. Currently, analyses of stock price movements typically focus on individual stocks or related stocks, with the principle that identifying relevance means seizing opportunities [2]. Consequently, current research methods on stock relationships primarily rely on statistical theory, establishing linear models between stock characteristics and prices. Early-stage methods include the Moving Average method [3] and Exponential Smoothing method [4], while later-stage methods encompass the Auto-regression model [5], Moving Average model, and the ARIMA model [6]. These methods have stringent requirements for data distribution and completeness, assuming a purely linear relationship among factors influencing stock prices. However, financial markets are influenced by the overall economy, industry characteristics, politics, and investor psychology [7]. Therefore, stock market changes are dynamic and chaotic [8]. Simple time series analyses can no longer accurately predict stock market changes. Developing models that can accurately predict stock market changes has been a key research focus for theorists and practitioners in recent years [9].

In recent years, with the advancement of machine learning technology, many researchers have begun using association mining to study economic markets and production management. Association mining involves identifying frequent patterns, associations, correlations, or causal structures among item sets in transaction data [10]. For example, Xue et al. (2019) utilized association mining to analyze the relationship between consumers' repeated product purchases [11]. Zhou et al. (2020) combined the MapReduce distributed computing model with association mining to propose a detection algorithm for medical insurance fraud based on frequent pattern mining [12]. Current research indicates that association mining algorithms can be widely applied across various fields, extracting relevant data from large, disordered datasets. As previously mentioned, financial markets are influenced by the overall economy and industry characteristics. Therefore, it is possible to identify relevant stocks from extensive data for stock price correlation analysis, providing decision-making references for stock market investment behavior. Compared to traditional artificial intelligence technology, the analysis and prediction of single stock time series may offer higher reliability [13]. Currently, most stock analyses using association rules focus on identifying related stock markets or industries [[14], [15], [16]], without considering whether these rules can provide future investment suggestions for stock market movements. Particularly, stock markets in different countries or regions have inconsistent opening and closing times. Therefore, our study explores whether the relationship between stock markets with different closing times can be analyzed through association rules, providing investors with a reference for the next day's trading. Although the algorithm we used is currently well-developed, the problem we address is relatively novel, representing the main innovation of this study.

This study employs association mining technology to determine the association and substitution rules between various stock markets, identifying stock price movement relationships to inform investment decisions, and thereby establishing an optimal AI model for stock investment decisions. Due to the time difference, the Taiwan stock market closes at 1:30 p.m., while the Chinese mainland and other international stock markets close later. After the Taiwan stock market closes, the closing prices of other international stock markets often serve as reference indices for Taiwanese investors on the next trading day. Therefore, we provide investors with purchase decisions by analyzing the correlation between international stock market movements and the Taiwan stock market the following day. Long-term testing of this method has shown high accuracy, ease of operation, and suitability for widespread use.

The selection of stock market samples primarily references Taiwan's economic and trade statistics. According to trade statistics from Taiwan's Ministry of Finance, Taiwan's current export targets include mainland China, India, Malaysia, Indonesia, the Philippines, Vietnam, Thailand, Germany, the United States, and other countries. This indicates that Taiwan has significant capital flow relationships with mainland China and other countries [17]. Thus, to maximize profits or avoid risks, Taiwan stock market investors must consider the performance of mainland and international stock markets. Accordingly, the major stock market indexes in mainland China and internationally, as well as the top 50 listed companies by market capitalization in the Taiwan securities market (referred to as Taiwan's 50 constituent stocks), were used as research subjects, with data from 743 trading days serving as historical reference. Considering the abnormal fluctuations caused by the global COVID-19 pandemic at the end of 2019, data from October 1, 2014, to October 31, 2018, were selected for this study. This study also analyzes the linkage between individual stocks to establish the application of association mining technology in stock market analysis, providing investors with references for stock price investment in the Taiwan stock market. Therefore, the main objective of this study is to determine the effectiveness of association mining in analyzing the correlation between stock market fluctuations, and through this method, to find the Taiwan and international stock markets with the highest degree of correlation for investors' reference.

The first chapter of this paper is the introduction, which introduces the research background, current research progress, and research objectives. The second chapter introduces relevant research concepts. The third chapter discusses our research methodology and data sources. The fourth chapter, we analyze stock data to identify the most relevant stocks. The final chapter provides a summary of the study, including conclusions, contributions, future research directions, and algorithm limitations.

## 2 Literature review

### 2.1 Association rule analysis

Initially, the association rule algorithm was applied to Market Basket Analysis in large stores. Namely, assuming that a customer buys product X and also buys product Y, thus forming an association between X and Y. Based on extensive calculations, interesting relationships within large datasets could be revealed through association rules or as collections of high-frequency items [18]. Among them, the most commonly used example to illustrate the association rule algorithm was the renowned Walmart supermarket in the American retail industry. Using association rule analysis, the relationships between purchased goods were analyzed from vast amounts of daily transaction data [19]. Currently, the application of association rule analysis has matured. For instance, Chen, Liu, and Liu (2013) used the Apriori algorithm to analyze the association between cultural consumer products and resident characteristics, It was found that cultural consumption habits varied between unmarried men and women [20]. In research on applying association algorithms to the stock market, Peng and Liu (2016) used the Apriori algorithm to study the rotation phenomenon in the Chinese stock market industry, establishing simple investment strategies based on strong association rules, which significantly outperformed the CSI300 index and industry benchmarks [21]. In Tsai (2012) research, the Association Rule technique in data mining was used to explore the associations between natural resource types of funds [22]. Using support, confidence, and gain values as thresholds, useful association rules were explored, and portfolio suggestions in line with public investment preferences were proposed, expected to offer investors different investment perspectives. Additionally, Manojlović and Štajduhar (2015) designed three groups of natural resource portfolio funds from the perspective of enterprises, providing references for commodity development or investment decisions [23].

### 2.2 Main algorithm of association rule mining

The traditional association rule algorithm currently consists of the Apriori algorithm, FP-growth algorithm, and Eclat algorithm. The core idea of the Apriori algorithm is the Apriori Principle, which states that if an item set is frequent, then all its subsets must also be frequent. Conversely, if an item set is non-frequent, then all its supersets must also be non-frequent [24]. The FP-growth algorithm uses a depth-first search method. First, by scanning the database, the transaction data is stored in a tree structure called the FP-tree. Then, by scanning the tree, the frequent patterns are identified. Using the algorithm, after identifying the frequent item sets ending in E, B, C, D, and A in turn and examining the path containing a specific node, the frequent item sets ending in E could be found [25]. Both the Apriori and FP-growth algorithms use a horizontal data format to mine frequent patterns from the transaction set in the TID-item set format (i.e., {TID: item_set}). The Eclat algorithm uses a vertical data format to mine frequent patterns from the transaction set in the item set-TID format (i.e., {item: TID_set}). First, the union of two frequent k-item sets is obtained to form the candidate k+1 item set. Then, the transaction sets of candidate k+1 item sets are intersected to generate frequent k+1 item sets. Finally, this process is iterated until the item set is normalized [26].

### 2.3 Application of association rule mining in stock market analysis

In current stock market research, most scholars study market fluctuations by establishing financial models. Research on alternative relationships in related stock markets using data mining methods is rare. For example, Huang, Liao, and Wang (2023) used different regression analysis models to predict the impact of international crude oil and gold futures price fluctuations on the Taiwan stock market and compared the advantages and disadvantages of various models [27]. Qiao, Dai, and Zhu (2017) used historical return backtesting and Granger causality tests to study the effect of A + H cross-listing on stock price movements [28]. They found a strong association between A + H stocks. Over time, the influence of A shares on H shares has increased significantly. In recent years, the application of association rule technology in stock market analysis has gradually increased, mainly used to reveal the correlation between different stocks. For example, Paranjape-Voditel and Deshpande (2013) propose a stock market portfolio recommender system based on association rule mining (ARM) that analyzes stock data and suggests a ranked basket of stocks [29]. Kartal et al. (2022) studied the co movement of global stock market indices, using association rule mining to reveal the synchronous relationship between markets and provide decision support for investors [30]. This study uses data mining association rules to analyze how the international stock market influences the rise and fall of the Taiwan stock market the following day. The goal is to provide investors with investment references for the Taiwan stock market.

## 3 Data sources and research methods

Data mining was conducted using SPSS Clementine (Trial Version). The software provided various data mining models, including neural networks, sequences, cluster analysis, and association rules. Association rule analysis was the primary modeling tool used in this data mining, with indicators mainly including Support, Confidence, and Lift.

Support primarily represents the probability of an event. Specifically, the support of formula (1) A→B refers to the proportion of events containing both event A and event B, that is, the probability that a change in stock market A and a change in stock market B will occur simultaneously.

(1) *Support(A→B)=fsup(A→B)=P(A ∩ B)*

Confidence represents the probability that event B will occur given that event A has already occurred in the dataset, as shown in formula (2). That is, the probability of a change in stock market B given a change in stock market A.

(2) $$confidence\left(A\to B\right)=fconf\left(A\to B\right)=\frac{P\left(A\cap B\right)}{P\left(A\right)}$$

If both formula (1) and formula (2) meet the minimum thresholds set, then A→B is considered to have a strong association.

Lift represents the ratio of the probability of both A and B occurring together to the probability of B occurring given A, as shown in formula (3). That is, in the case of a change in stock market A, it includes the change in stock market B, and only the ratio of the change in stock market B.

(3) $$lift\left(A\to B\right)=P\frac{P\left(A\cap B\right)}{P\left(A\right)P\left(B\right)}$$

**Fig. 1:**
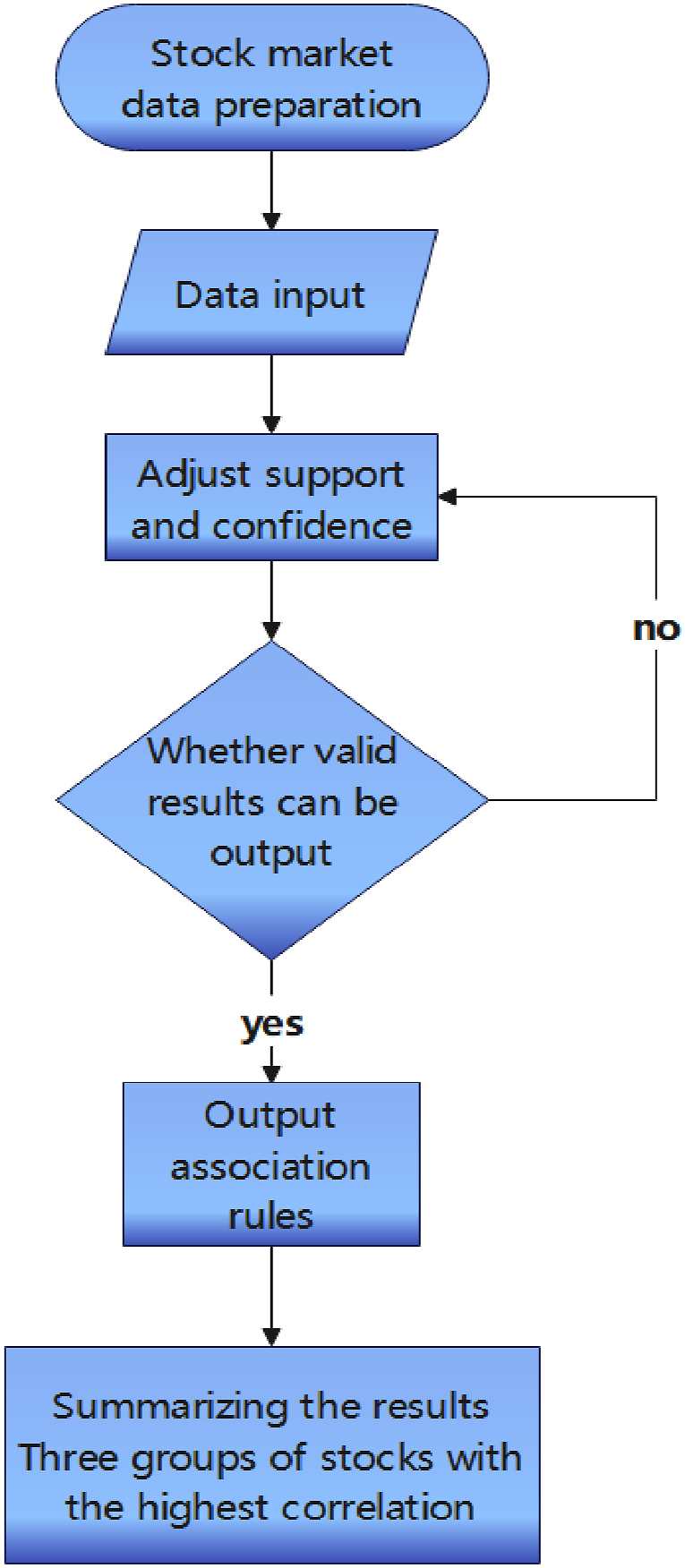
Research process.

Currently, the association rule algorithm mainly comprises the Apriori algorithm, FP-growth algorithm, and Eclat algorithm. To find the best algorithm for stock market analysis, we first assessed the effectiveness of the Apriori, FP-growth, and Eclat algorithms, then analyzed the overall sample. In terms of results, setting support and confidence levels too high may not yield any association rules, while setting them too low may produce too many results. Referring to previous studies, we started with a minimum support of 20 % and a minimum confidence of 55 %, gradually mining to obtain the most suitable stock portfolio for investors' reference. An overly extensive portfolio may interfere with decision-making. Therefore, we limited the frequent item sets to 3 groups to identify the stock portfolio with the highest support and confidence. The research framework is illustrated in the following Fig. 1.

The research data were derived from the Taiwan Economic Journal database (TEJ+) and the Yahoo Stock Market. All our data are uploaded to Heliyon journal as attachments. This information included the closing times and prices of major stock market indexes in the Americas, Asia, and Europe, as well as Taiwan's 50 constituent stocks.

The stock price information of Taiwan's 50 Constituent Stocks and the Mainland and International Stock Market Indexes was sorted separately for each trading day and categorized as rise, fall, or flat. The specific method was as follows: the closing prices of the previous and subsequent trading days were subtracted. If the resulting value was positive, it indicated a rise; if negative, a fall; and if zero, it was flat. Additionally, considering that the stock markets of the countries from which the data were collected had different national holidays, if any stock market was not trading on a given day, all data for that day were excluded. After processing, a total of 743 transaction data points were obtained.

The main purpose of this research was to explore the association between stock market indexes in the United States, Europe, and Asia and Taiwan's 50 constituent stocks. Differences in closing times between countries were considered. The closing time of the Taiwan stock market is 1:30 p.m., while the closing times of mainland China and other international stock markets are later. Moreover, since the closing prices of various countries often serve as reference indices for Taiwanese investors on the next trading day, the closing date of Taiwan's 50 constituent stocks is aligned with the closing date of other international stock market indexes the following day. The specific international stock markets and their closing times are shown in Table 1 below.

**Table 1:** The international stock markets included in this research.

	Name	Closing time
Asia	Nikkei Index	Beijing Time: 14:15pm
	KOSDAQ	Beijing Time: 14:00pm
	Straits Times Index	Beijing Time: 17:00pm
	Malaysia Composite Index	Beijing Time: 17:00pm
	The Australian Stock Index	Beijing Time: 14:00pm
	India SENSEX Index	Beijing Time: 17:30pm
United States, Canada	DJIA (DowJones Industrial Average Index)	Beijing Time: +1 04:00am
	Nasdaq Composite Index	Beijing Time: +1 05:00am
	S&P 500 Index	Beijing Time: +1 04:00am
	PHLX Semiconductor Sector	Beijing Time: +1 05:00am
	Russell 2000 Index	Beijing Time: +1 05:00am
	Canadian Composite Index	Beijing Time: +1 06:00am
Europe	French CAC Index	Beijing Time: +1 00:00am
	German DAX index	Beijing Time: +1 02:00am
	Financial Times Index	Beijing Time: +1 00:00am

## 4 Result analysis

In the results analysis section, the running time and the number of rules obtained by different algorithms were first analyzed to explore the differences in stock market association analysis. Then, the association rules between the Taiwan stock market and the international stock market were analyzed by gradually increasing the support and confidence to identify the rules with the strongest associations.

### 4.1 Comparative analysis of association rule algorithm

The purpose of the comparative analysis was to demonstrate the effectiveness of different association rule algorithms in stock market forecasting. In the comparative analysis, 2000 sets of stock market data were selected, with a minimum support set at 20 %. The accuracy and calculation speed of the three different algorithms were compared, as shown in Table 2 below.

**Table 2:** Differences in results of different algorithm runs.

Suppor	Apriori	FP-growth	Eclat	Consequent	Antecedent	Confidence
	Number/Time	Number/Time	Number/Time			
20 %	10142/0.825	15320/101.214	4104/1.015	2330 TSMC =Fall	Canadian Composite Index = Fall	57.333
					Russell 2000 Index = Fall	
					S&P 500 = Fall	
					Financial Times Index = Fall	
25 %	2115/0.241	1991/22.167	1026/0.512	2330 TSMC =Fall	Canadian Composite Index = Fall	59.585
					Nasdaq Composite Index = Fall	
					Russell 2000 Index = Fall	
30 %	815/0.018	206/2.157	185/0.025	2317 Hitech = Rise	Shanghai A-share Index = Rise	57.585
					Financial Times Index = Rise	
35 %	26/0.016	0/0	26/0.016	2454 MTK+1 = Fall	Financial Times Index = Fall	53.558
					French CAC index = Fall	
40 %	19/0.016	0/0	19/0.016	4938 Pegatron +1 = Rise	Korea Composite Index = Rise	53.453

The comparison of the number of rules generated by different algorithms in Table 2 reveals that the Apriori algorithm generates more rules than the other two algorithms under high support parameters. Due to the large number of generated rules, only the association rules with the highest confidence under different support levels are listed. In the subsequent study, the three algorithms will be used to analyze the overall stock data.

### 4.2 Association mining analysis results

In the association mining, parameters were set according to different confidence and support conditions, and the number of different Apriori rules was generated accordingly. Smaller parameter values yield more results, but the reference value of these results decreases accordingly. Conversely, higher parameter values yield fewer results, but the reference value of these results is higher. Drawing on previous research, starting with a minimum support level of 20 % and a minimum confidence level of 55 % yielded more easily obtainable experimental results (Huang & Li, 2018). The minimum support thresholds were set to 20 %, 25 %, and 30 %, representing the percentage of the total number of transactions; the minimum confidence thresholds were set to 55 %, 60 %, and 65 %, representing the percentage of trading days meeting the antecedent conditions. The maximum number of antecedents was limited to four items.

Association mining was applied to explore the linkage between mainland and international stock market indexes and Taiwan's 50 constituent stocks on the following day. Various combinations of 21 mainland and international stock market indexes and 48 of Taiwan's 50 constituent stocks revealed stocks with high associations between rises and falls. The research data consisted of 51,336 trading records from the included stock market indices and stocks over 744 days between October 1, 2014, and October 31, 2018. By varying the minimum antecedent support and minimum rule confidence, performing calculations, and comparing the emerging rules, the research aimed to explore optimal association rules as investment references for investors. The maximum number of antecedents was set to four, with a minimum support of more than 25 % and confidence of more than 60 %. The rules generated after the operation are explained in the table below. Among them, Rule ID 359 indicated that when the Hang Seng Index (HSI), the S&P 500 Index, the Dow Jones Industrial Index, and the PHLX Semiconductor Sector (SOX) rose, accounting for about 25.13 % of the total data transactions on the trading day, TSMC's (2330) confidence rose by 62.57 % the next day. Rule 11 implied that when the Canadian Composite Index and the Dow Jones Industrial Index fell together, accounting for about 30.51 % of the total trading day data, Asian Cement's (1102) confidence fell by 62.56 % the next day. Table 3 provides the details.

**Table 3:** Association results of minimum support of 25 % and minimum confidence of 60 %.

Consequent	Antecedent	Rule ID	Support	Confidence
2330 TSMC =Rise	Hang Seng Index (HSI) =Rise	359	25.134	62.567
	S&P 500 = Rise			
	Dow Jones Industrial Index = Rise			
	PHLX Semiconductor Sector (SOX) = Rise			
2330 TSMC =Rise	S&P 500 = Rise	366	25.134	62.567
	Dow Jones Industrial Index = Rise			
	Shanghai Stock Exchange B-share Index = Rise			
	Nasdaq Composite Index = Rise			
1303 South Asian stock = Rise	Hong Kong Hang Seng Index (HSI) =Rise	395	25.134	62.567
	S&P 500 = Rise			
	Dow Jones Industrial Index = Rise			
	PHLX Semiconductor Sector (SOX) = Rise			
1102 Asian cement = Fall	Canadian Composite Index = Fall	11	30.551	62.555
	Dow Jones Industrial Index = Fall			
4938 Pegatron stock = Rise	French CAC index = Rise	275	25.806	62.5
	Financial Times Index = Rise			
	Dow Jones Industrial Index = Rise			
	Canadian Composite Index = Rise			
4938 Pegatron stock = Rise	French CAC index = Rise	278	25.403	62.434
	Financial Times Index = Rise			
	Dow Jones Industrial Index = Rise			
Algorithm	Apriori	FP-growth		Eclat
Time	2.482	1.9420		2.0126

According to Table 3, there are a total of six association rules at a minimum support level of 25 % and a minimum confidence level of 60 %, which could serve as references for investors in Taiwan's stock market. From the perspective of algorithm running time, FP-growth has the shortest running time among the three association rule algorithms, taking only 1.9420 s. In the follow-up, to mine more association rules, the support level of the association mining rules was reduced to 20 % and the confidence level was increased to 65 %. This means that on 20 % of trading days, there was 65 % confidence that prices would rise or fall simultaneously. The association rules at a minimum support level of 20 % and a minimum confidence level of 65 % are shown in Table 4 below. Due to space constraints, only the highly associated rules have been listed.

**Table 4:** Association results of minimum support of 20 % and minimum confidence of 65 %.

Consequent	Antecedent	Rule ID	Support %	Confidence %
2354 Foxconn Stock = Rise	French CAC index = Rise	21	21.102	66.879
	Financial Times Index = Rise			
	Hong Kong Hang Seng Index (HSI) =Rise			
	PHLX Semiconductor Sector (SOX) = Rise			
	Russell 2000 Index = Rise			
2330 TSMC =Rise	Canadian Composite Index = Rise	9	21.774	65.432
	Shanghai A-share Index = Rise			
	Nasdaq Composite Index = Rise			
2330 TSMC =Rise	Russell 2000 Index = Rise	10	21.774	65.432
	Canadian Composite Index = Rise			
	Shanghai Securities Composite Index (SSEC) =Rise			
	Nasdaq Composite Index = Rise			
1303 South Asian stock = Rise	Dow Jones Industrial Index = Rise	15	20.027	65.101
	Shanghai B-share index = Rise			
	Canadian Composite Index = Rise			
	Nasdaq Composite Index = Rise			
2317 Hitech = Fall	PHLX Semiconductor Sector (SOX) = Fall	5	21.505	65.000
	Canadian Composite Index = Fall			
	Russell 2000 Index = Fall			
	S&P 500 Index = Fall			
Algorithm	Apriori	FP-growth		Eclat
Time	11.7615	8.1608		9.2141

According to Table 4, with a minimum support level of 20 % and a minimum confidence level of 65 %, it was observed that 35 rules were generated. This indicates that under normal fluctuations of these mainland and international stock market indexes, the confidence of one of Taiwan's 50 constituent stocks would rise or fall by 65 % simultaneously the next day. Subsequently, the minimum support level and minimum confidence level were adjusted again. The calculation was then based on a minimum support level of 30 % and a minimum confidence level of 60 % to explore whether more significant association rules could be found, as shown in Table 5 below. Due to space constraints, only the rules with a high degree of association are listed.

**Table 5:** Association results of minimum support of 30 % and minimum confidence of 60 %.

Consequent	Antecedent	Rule ID	Support %	Confidence %	Lift
2354 Foxconn Stock+1 = Rise	French CAC index = Rise	52	30.376	62.832	1.309
	Financial Times Index = Rise				
	PHLX Semiconductor Sector (SOX) = Rise				
1102 Asian cement+1 = Fall	Canadian Composite Index = Fall	3	30.511	62.555	1.318
	Dow Jones Industrial Index = Fall				
2330 TSMC+1 = Rise	Russell 2000 Index = Rise	72	31.720	62.288	1.313
	Dow Jones Industrial Index = Rise				
	Canadian Composite Index = Rise				
	Nasdaq Composite Index = Rise				
4938 Pegatron stock +1 = Rise	Financial Times Index = Rise	60	31.183	61.638	1.303
	S&P 500 Index = Rise				
	Dow Jones Industrial Index = Rise				
	Nasdaq Composite Index = Rise				
2354 Foxconn Stock+1 = Rise	French CAC index = Rise	51	32.124	61.506	1.282
	Financial Times Index = Rise				
	Nasdaq Composite Index = Rise				
Algorithm	Apriori	FP-growth		Eclat	
Time	0.5052	0.5516		0.6021	

Table 5 shows that with a minimum support level greater than 30 % and a minimum confidence level greater than 60 %, 76 rules were generated. This indicates that under the general ups and downs of these international stock market indexes, the confidence of one of Taiwan's 50 constituent stocks would rise or fall simultaneously by 60 % the next day. Regarding algorithm running time, increasing the minimum support resulted in the Apriori algorithm shortening its running time, making it the fastest among the three association rule algorithms, with a running time of only 0.5052 s. Subsequently, the study adjusted the minimum support and minimum confidence again, setting the minimum support at 30 % and the minimum confidence at 55 % for the calculations. The aim was to determine if more meaningful association rules could be found. The specific results are shown in Table 6. Due to space constraints, only the rules with higher correlation have been listed.

**Table 6:** Association results of minimum support of 30 % and the minimum confidence of 55 %.

Consequent	Antecedent	Rule ID	Support %	Confidence %	Lift
2330 TSMC+1 = Rise	Russell 2000 Index = Rise	2101	31.720	62.288	1.313
	Dow Jones Industrial Index = Rise				
	Canadian Composite Index = Rise				
	Nasdaq Composite Index = Rise				
2354 Foxconn Stock+1 = Rise	French CAC index = Rise	2147	30.108	61.161	1.275
	Financial Times Index = Rise				
	German DAX index = Rise				
	Nasdaq Composite Index = Rise				
2354 Foxconn Stock+1 = Fall	PHLX Semiconductor Sector (SOX) = Fall	906	30.914	60.870	1.294
	Nasdaq Composite Index = Fall				
	S&P 500 Index = Fall				
2454 MTK +1 = Fall	PHLX Semiconductor Sector (SOX) = Fall	106	30.108	60.268	1.263
	Dow Jones Industrial Index = Fall				
2308 Delta +1 = Fall	Nasdaq Composite Index = Fall	916	32.661	60.082	1.315
	Russell 2000 Index = Fall				
	S&P 500 Index = Fall				
Algorithm	Apriori	FP-growth		Eclat	
Time	0.4866	0.5459		0.5725	

When the minimum support is greater than 30 % and the minimum confidence is greater than 55 %, there are 2202 rules. This means that under the general fluctuations of these international stock market indexes, one of Taiwan's 50 constituent stocks will have a 55 % chance to rise or fall simultaneously the next day. Regarding algorithm running time, the Apriori algorithm has the shortest running time among the three association rule algorithms, taking only 0.4866 s.

### 4.3 The stock market with the highest degree of association

In this research, focusing on the association between the mainland Chinese stock market and the international stock market index on the next day of Taiwan's 50 constituent stocks, the antecedent and consequence data were compiled based on the maximum confidence of the aforementioned three groups, as illustrated in Table 7 below.

**Table 7:** Table of Antecedent and Consequence of the first three groups with the maximum confidence.

Support %	Confidence %	Lift	Antecedent	Consequent
21.102	66.879	1.394	French CAC index = Rise	2354 Foxconn Stock+1 = Rise
			The Financial Times Index = Rise	
			Hang Seng Index (HSI) =Rise	
			PHLX Semiconductor Sector (SOX) = Rise	
30.376	62.832	1.309	French CAC index = Rise	2354 Foxconn Stock+1 = Rise
			The Financial Times Index rose	
			PHLX Semiconductor Sector (SOX) = Rise	
31.720 %	62.288 %	1.313	Russell 2000 Index = Rise	2330 TSMC+1 = Rise
			Dow Jones Industrial Index = Rise	
			Canadian Composite Index = Rise	
			Nasdaq Composite Index = Rise	

According to the highest association model in Tables 7 and it can be analyzed that when the French CAC index, the British Financial Times Index, the Hang Seng Index (HSI), and the Philadelphia Semiconductor Index all rose simultaneously (accounting for 21.102 % of all trading days), Foxconn Stock (2354) would rise by 66.879 %. (The second and third groups of explanations were similar.) Consequently, the analysis of the association rules was expected to greatly improve the accuracy of predicting stock market movements, aiding in stock market investment.

Our paper investigates the correlation between international stock markets, although previous studies have focused on correlation analysis within stock markets. However, most studies have only aimed at finding related stock markets within the same market. Our research innovatively considers the situation where international stock markets have different closing times and analyzes the correlation between the closing prices of the same day's stock market and the next day's stock market. This aims to use the results of association rules as a reference for next day's stock market investment, ensuring high reliability. This is the main innovation of this study.

### 4.4 Robustness test

The most commonly used method for robustness testing was to transform the original sample spacing, as different sample selection periods may have different effects on the results. In Lim et al. (2019) research, the sample spacing was evenly divided into two parts for regression [31]. It was found that only the regression results of the latter period were similar to the original conclusion, while the sample was not significant for a period of time. Therefore, to explore the association between stock markets in different periods, the method of transforming the sample spacing was adopted. The sample interval was divided into two parts: from October 1, 2014, to October 15, 2016, and from October 16, 2016, to October 31, 2018. From the results of the robustness test, it was inferred that regardless of whether the sample interval was between October 1, 2014, to October 15, 2016, or October 16, 2016, to October 31, 2018, the first three groups with the highest reliability were the stock market combinations shown in Table 7, thereby proving the robustness of this model's results.

## 5 Conclusion

This research demonstrated that through the analysis of association rules, the association between stock markets in various countries could be explored. Based on the associated stocks and stock markets identified in the algorithm, particularly, the Taiwan stock market was more closely related to the top ten economies such as mainland China, the United States, the United Kingdom, and France. This implied that a rise in international or mainland stock markets would prompt foreign investors to actively overbuy Taiwan's stock market, and vice versa. This result is also in line with previous research on the substitution relationship of related industries in the stock market [[32], [33], [34]]. At the same time, we also verified the substitution effect of Taiwan's industrial structure and international industries proposed by Simon [35]. That is to say, Taiwan's science and technology industry has a great reputation worldwide. When a country's science and technology industry becomes a hot topic, Taiwan's science and technology industry also receives attention. Therefore, analyzing and predicting the stock market through association rules is effective. Furthermore, from the perspective of the stock industry structure of the model analysis results, Taiwan is known for producing and exporting semiconductors and original equipment manufacturing (OEM) of electronic-related parts. There is a strong association between the rise and fall of stock prices in Taiwan's semiconductor industry (such as TSMC) and electronics industry (such as Hitech and Foxconn) and foreign customers. Thus, once technology stocks become a popular theme in a certain country (namely, the United States), Taiwan's related industries are noticed by foreign investors, and the stock price is naturally affected.

The main contribution of this study is to propose a new method to analyze and effectively predict the rise and fall of stocks. Our research analyzes the association rules of massive stock trading data and finds that the use of association rules can identify the substitution relationship in the relevant stock market and yield good results. This enables us to predict the rise and fall of the Taiwan stock market the next day based on this correlation. Additionally, we compared the differences among the three association mining algorithms in stock market analysis and found that the FP-growth algorithm runs the fastest when set with low support and high confidence. After improving the support and reducing the confidence, the operation speed of the Apriori algorithm becomes faster. This is an interesting phenomenon. In the future, we can conduct in-depth research on the differences among the three algorithms to explore the causes of this phenomenon. Finally, this study has some limitations. Because the association rule algorithm is only suitable for mining single transactions, the study uses the closing price as a sample for analysis. Many factors affect the stock market. If we consider the closing price, opening price, investor sentiment, and other related factors, the association rule algorithm may not be suitable for analysis.

## CRediT authorship contribution statement

**Luote Dai:** Writing – review & editing, Writing – original draft, Software, Methodology, Investigation, Conceptualization. **Chengkui Huang:** Writing – review & editing, Methodology, Investigation. **Chuyu Yu:** Writing – review & editing, Writing – original draft, Methodology. **Shengyu Gu:** Writing – review & editing, Writing – original draft.

## Declaration of competing interest

The authors declare that they have no known competing financial interests or personal relationships that could have appeared to influence the work reported in this paper.
